# Follow the leader? Orange-fronted conures eavesdrop on conspecific vocal performance and utilise it in social decisions

**DOI:** 10.1371/journal.pone.0252374

**Published:** 2021-06-09

**Authors:** Heidi M. Thomsen, Thorsten J. S. Balsby, Torben Dabelsteen

**Affiliations:** 1 Department of Biology, University of Copenhagen, Copenhagen, Denmark; 2 Department of Bioscience, Wildlife Ecology, Aarhus University, Rønde, Denmark; University of Missouri Columbia, UNITED STATES

## Abstract

Animals regularly use social information to make fitness-relevant decisions. Particularly in social interactions, social information can reduce uncertainty about the relative quality of conspecifics, thus optimising decisions on with whom and how to interact. One important resource for individuals living in social environments is the production of information by signalling conspecifics. Recent research has suggested that some species of parrots engage in affiliative contact call matching and that these interactions may be available to conspecific unintended receivers. However, it remains unclear what information third parties may gain from contact call matching and how it can be utilised during flock decisions. Here, using a combined choice and playback experiment, we investigated the flock fusion choices and vocal behaviour of a social parrot species, the orange-fronted conure (*Eupsittula canicularis*), to a contact call matching interaction between two individuals of different sexes and with different vocal roles. Our results revealed that orange-fronted conures chose to follow vocal leaders more often than vocal followers during fusions. Furthermore, flocks responded with higher call rates and matched the stimulus calls closer when subsequently choosing a vocal leader. Interestingly, orange-fronted conures also showed higher contact call rates and closer matches when choosing males over females. These results suggest that paying attention to conspecific contact call interactions can provide individuals with social information that can be utilised during fission and fusion events, significantly influencing the social dynamics of orange-fronted conures.

## Introduction

Animals continuously seek information about their environment to reduce uncertainty and optimise decision-making [1–3]. As opposed to information acquired from direct interaction with the environment (personal information), cues and signals from other individuals (social information) can be obtained at reduced costs and provide additional information that can lead to more accurate estimates of environmental parameters [1, 4, 5]. As a result, animals regularly use social information to inform fitness-relevant decisions, including mate choice and breeding- or foraging site selection [6–8]. One particularly important resource for individuals living in social environments is the production of information by signalling conspecifics. In many communication systems, signalling interactions are conspicuous and can be observed by bystanders [9]. In these systems, individuals that are not the intended receivers can extract information from signallers by eavesdropping on their interactions [9, 10]. Eavesdropping can be beneficial for decision-making in a variety of ecological contexts [e.g. 11–15]. Particularly in social interactions, competitive as well as collaborative, social information can reduce uncertainty about the relative quality of conspecifics, thus optimising decisions on with whom to interact [e.g. 12, 14, 16, 17].

Vocal signals often travel over much larger distances than the average spacing between individuals, making vocal interactions an important informational resource prone to be eavesdropped upon by conspecifics [9, 18, 19]. For instance, most species of songbirds have a repertoire of functionally equivalent song types [20]. During aggressive singing interactions, individuals may vocally match some aspect of the song (e.g. song type) of the other interactants [21, 22]. Song matching has been argued to be a signal of aggressive intent used by males to establish and maintain territory boundaries during the breeding season [23, 24]. Furthermore, song matching may convey useful information for listening individuals as it allows for a direct comparison of performance quality or motivation for male and female eavesdroppers [21, 25–28]. For example, [29] found that listening male great tits (*Parus major*) discriminate between individuals depending on their patterns-specific singing performance by modifying their behaviour in subsequent encounters with the interactants. Other examples involve the use of relative information gathered by eavesdropping in the mating decision of females [e.g. 14, 30] and song learning in juvenile birds [31].

Although vocal matching seems to be a common phenomenon, its utility for third parties has only been studied in a few species [32]. Matching of conspecifics’ individual-specific vocalisations has also been shown during interactions in parrots [33–35]. Parrots are known to immediately match contact calls of other interactants by changing the acoustic structure of their individually distinctive calls during vocal exchanges [33, 36]. Over the course of the interaction, an individual’s contact calls may gradually become more similar to the contact calls of another individual, resulting in a convergent exchange of contact calls [37]. Whereas songbirds are often limited to matching individuals with whom they share existing song types [e.g. 21, 25], parrots can match contact calls from individuals with little or no prior learning [33–35]. Such rapid vocal matching is hypothesised to precede and mediate the formation of groups where individuals live in social systems with insufficient compositional stability to support group-specific calls [38–40]. However, empirical studies on responses of third parties towards contact call matching are scarce. Testing the extent to which vocal matching influences the behaviour of unintended conspecific listeners has been difficult owing to challenges in conducting experiments with wild parrots and controlling or quantifying the social information available during their vocal interactions. In this study, we aimed to reduce this knowledge gap by systematically testing for an effect of vocal behaviour during contact call matching on third party flock decisions in a social parrot, the orange-fronted conure (*Eupsittula canicularis*).

Orange-fronted conures live in a fission-fusion social system in which individuals travel and forage in flocks characterised by frequent changes in composition during the non-breeding season [39, 41]. During the non-breeding season, orange-fronted conures often form large night roost aggregations that will break up into smaller foraging flocks at dawn. Although some local variation occurs, such foraging flocks typically consist of 1–6 individuals [41, 42]. During vocal interactions, orange-fronted conures may modify the fine-scale structure of their contact calls to match contact calls of other interactants prior to flock fusions [34, 43], suggesting that contact call matching has an affiliative function [37] and may facilitate the direction of signals to specific individuals (addressing) in a flock [44]. Furthermore, performance roles (matching vs not matching) during vocal interactions [36] may reflect differences in individuals’ motivation, social status or propensity to be cooperative [37, 45]. Orange-fronted conures provide a particularly interesting system to examine social information use for at least two reasons. Firstly, contact call exchanges always precede decisions to merge or split during flock encounters, suggesting that the information exchanged within a single vocal interaction is sufficient to decide whether or not to fuse [41]. The social and physical environment limits contact through other modalities, and discrimination of individuals is suggested to be facilitated by using individually specific contact calls [36, 44]. Thus, orange-fronted conures provide a valuable opportunity to directly manipulate available social information and its use prior to flock fusion decisions. Secondly, there will often be several flocks within contact calling range of each other in an area [41], which provides ample opportunity for eavesdropping. Individuals living in dynamic flocks, such as the fission-fusion social systems of many parrots, are often exposed to a large number of conspecifics [46], making acquiring and maintaining accurate information about their social environment by direct interaction unfeasible. Under such circumstances, social information embodied by the contact call matching interactions could prove valuable for listeners when deciding whom to associate with [36]. Parrots have been observed to engage in a limited number of cooperative acts in the wild [47–49], and laboratory experiments have demonstrated that parrots have the capacity to cooperate with conspecifics [50, 51]. Combined, the dynamic nature of orange-fronted conures’ social structure and the potential adaptive benefits of cooperation would favour the emergence of a system for inferring reputation and copying of social choices across a wide variety of contexts [52]. For instance, mate choice copying, where females increase relative preferences for males after seeing them preferred by other females, has been demonstrated in a number of different species [e.g. 53–55]. Such copying may be adaptive when the cost (e.g. time and energy) to evaluating the quality of an individual is high or when discriminating between the quality of individuals is difficult [56, 57]. More recent experiments have demonstrated similar effects of social learning in recognition of group members as cooperative or defective in primates [16] and domestic dogs [58]. Vocal matching has been shown to indicate affiliative intent in social species [44, 59], and individuals vocally matching conspecifics could thus be valuable in such decision copying. However, despite a growing research interest in affiliative vocal matching [32, 45, 59], it remains unclear what information unintended receivers may gain and utilise from eavesdropping on vocal matching interactions in parrots.

Here, we present a novel experimental design to test what information third parties may utilise from eavesdropping on affiliative contact call matching interactions and its potential importance in the fusion decisions of orange-fronted conure flocks. Extending studies of vocal matching between competitors to that between social affiliates will help expand the understanding of how third parties perceive vocal matching in general and determine how information from affiliative vocal interactions may affect the social dynamics of fission-fusion systems. To explore this, we conducted a field experiment, where we broadcasted simulations of vocal matching of contact calls between two interactants to wild orange-fronted conure flocks, presenting them with a choice to fuse with one of the interactants. Using a multi-speaker experimental design enabled us to simulate an asymmetrical interaction between two conspecifics where the contact calls from one interactant (leader) would always precede those of the other interactant (follower). By creating this asymmetry in timing, we were able to simulate a vocal matching of contact calls by the follower to those of the leader [34]. Thus, allowing us to systematically investigate the influence of simulated vocal roles and interactant sex on the fusion decision of eavesdropping conspecifics. Specifically, we tested if orange-fronted conures discriminate between simulated leaders and followers of different sexes when choosing which interactant to fuse with and how these choices affect their vocal responses during the decision process. Furthermore, we aimed to test to what extent this is affected by the size of flocks and the level of flock decision consensus. If orange-fronted conures discriminate between simulated interactants, we hypothesise that flocks may mitigate the limited personal information available by copying the affiliative behaviour shown by the simulated follower. If this is true, we expect focal flocks predominantly to fuse with the simulated vocal leader of the interactions.

## Materials and methods

We conducted the playback experiments in June-August 2015 and June-July 2016 at three sites located 7–11 km apart in Área de Conservación Guanacaste, Costa Rica: Centeno (10°52.67’N 85°34.40’W), Naranjo (10°50.11’N 85°37.47’W) and Santa Elena (10°56.15’N 85°35.61’W).

### Playback setup

**Fig 1:**
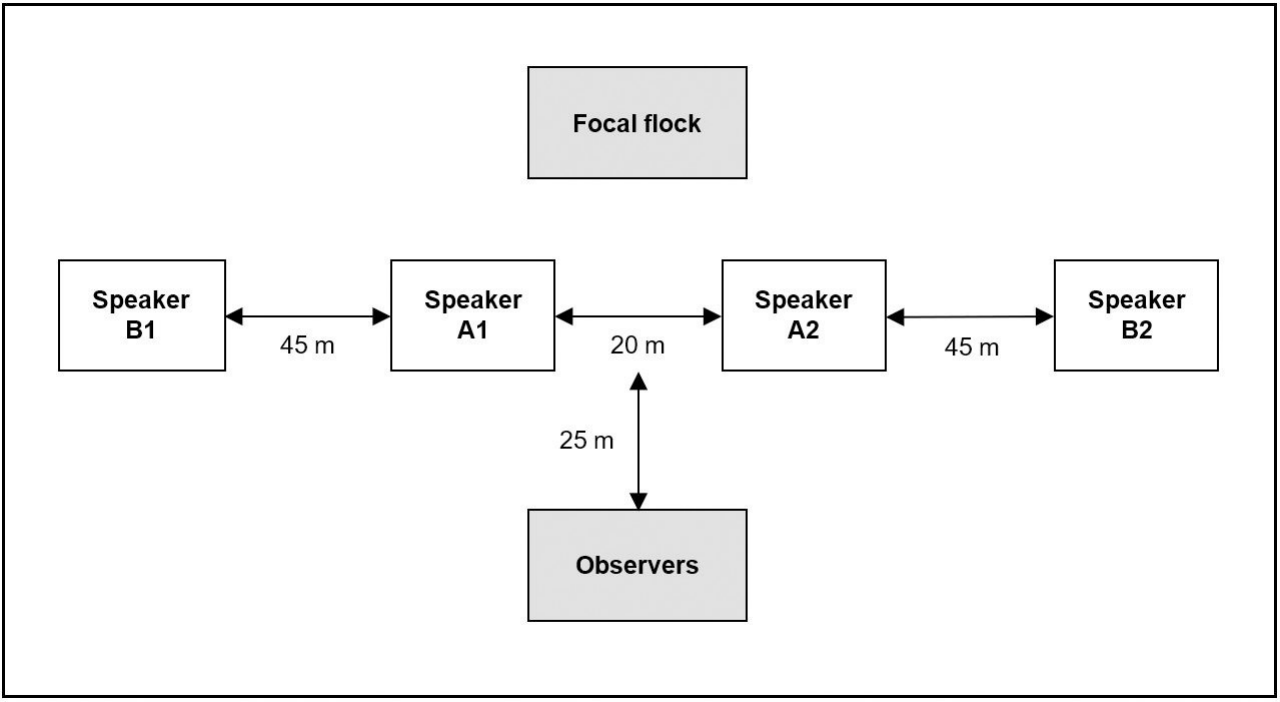
Schematic overview of the experimental speaker setup. Note that the schematic overview is not drawn to scale.

Each playback setup consisted of four JBL Control 1 Pro speakers connected to a Denon DCA-600 amplifier controlled by a computer (HP EliteBook 840 GI) using Audacity 2.1.0 (http://audacity.sourceforge.net/). We arranged the speakers in a straight line consisting of two close speakers (A1 and A2) 20 m apart and two remote speakers (B1 and B2) placed 45 m on each side of the close speakers (Fig 1).

The amplifier was adjusted to natural output sound pressure of 95 dB(A) at 1 m [39]. Observers would stay hidden approximately 25 m from the close speakers.

### Playback stimulus preparation

The contact calls used for playback stimuli were selected from an extensive library of individuals recorded in June-July 2005 and 2006. We selected 30 male and 10 female orange-fronted conures to provide the playback stimulus (stimulus individuals) by choosing the individuals with the highest number of contact calls. Their sex was determined molecularly from blood samples extracted from the wing vein prior to recording (primer descriptions in [60, 61]). The birds were held temporarily in aviaries and recorded using a Sennheiser MKH816 microphone and a Marantz PMD 670 hard disc recorder [62]. The stimulus individuals were caught within 10 km of the experimental sites, a distance equivalent to the typical foraging flights of orange-fronted conures during the non-breeding season [39]. They were thus considered geographically similar to those of our study population [43].

Each playback trial consisted of two parts: an interaction phase and a choice phase. The purpose of the interaction phase was to present orange-fronted conure flocks with an opportunity to extract information from a simulated contact call interaction between two stimulus individuals, where the interactants had distinct vocal roles. During the interaction, one stimulus individual consistently acted as the leader by calling first, followed by the other stimulus individual. We specifically tailored the playback stimuli so the contact calls of the follower and leader would gradually converge, i.e. become more similar and hence match each other (specified below) [34]. Fifteen seconds after the interaction phase, we presented the flock with the choice phase, in which the interactants would no longer have distinct vocal roles, although contact calls would remain converged.

We created interactions between two male stimulus individuals and a female and a male stimulus individual using AviSoft-SASLab Pro 5.2.12 (Avisoft Bioacoustics, Berlin, Germany). Similarities between contact calls of stimulus individuals were quantified by pairwise spectrographic cross-correlations (SPCC) (FFT length: 512 pts, overlap: 93.7%, Hann window, bandwidth: 500–11000 Hz) in MATLAB 7.1 (Mathworks, Natick, Massachusetts, USA) [63]. From each stimulus individual, we included a minimum of 90 contact calls in the correlations. All calls were filtered (0.5–11 kHz) and standardised with respect to peak amplitude prior to SPCC. For the interaction phase, we selected 15 contact calls from each interactant, of which five would show low levels of similarity (SPCC coefficients between 0.35 and 0.45), five showed intermediate levels of similarity (SPCC coefficients between 0.55 and 0.65) and five showed high levels of similarity (SPCC coefficients between 0.65 and 0.75). For the choice phase, we selected six contact calls from each interactant that showed high levels of similarity (SPCC coefficients over 0.70) that would be used twice, resulting in 12 stimulus calls. Throughout the experiment, each stimulus individual would act as leader of two and follower of two interactions but never with the same stimulus individual as the opponent in the interaction. Each stimulus contact call was only used in one interaction.

### Playback execution

Playbacks to wild flocks were carried out at 5:30–10:30 am and at 2:30–5:30 pm, which reflects periods of high vocal activity and hence increased likelihood of flocks responding. We rotated trials among the three sites so that at least one day elapsed between a return to the same site to reduce the chances of repeated interactions with the same flock. In total, we completed 22 successful male-male and 18 male-female trials at the Santa Elena, 6 successful male-male and 12 male-female trials at the Centeno and 12 successful male-male and 10 male-female trials at the Naranjo site.

The playback design required us to attract wild flocks of orange-fronted conures to the playback speakers. Five contact calls from either the leader or the follower of the interaction in each trial were played back to attract overflying flocks. Stimulus calls used as attraction were not part of the playback stimulus. Contact calls from all stimulus individuals were used as an attraction in one trial where they acted as a leader and one trial where they acted as a follower. The calls used as attraction were played interactively from either speaker A1 or A2 at 2–5 s intervals until a flock landed within 50 meters of the speakers. The use of speaker A1 and A2 in attracting flocks was balanced with regard to vocal role and sex of stimuli individuals. When a flock landed, the trial started. The trial would be chosen randomly from the pool of playbacks not previously used on the site. All playbacks were anonymised, ensuring that the identity of each stimulus individual would be unknown to the observers. Trials were aborted if multiple flocks were attracted, or focal flocks left before the first stimulus call in the choice phase of the playback.

The first stimulus call demarcated the start of the interaction phase. The trial’s interaction phase (average ± SE: 129 ± 1 s) was implemented by playing alternatingly from speaker A1 and A2 –one interactant in each speaker. The vocal roles were obtained by creating a two-channel playback in which the selected contact calls from each stimulus individual would be added to separate channels in a set pattern (S1 Table). Each stimulus call from the leader would be followed by a stimulus call from the follower after 1–3 s with a spacing of 6–10 s and 6–11 s between calls from the leader and follower. The timing of the vocal interactions in the playback reflects natural interactions between wild orange-fronted conures. During the interaction, the similarity between contact calls from the leader and the follower would gradually increase (S1 Table) to indicate vocal convergence [37, 62]. The interaction phase ended 15 s after the last stimulus call, at which point the choice phase started. The choice phase was created by moving the playback from speaker A1 and A2 to speaker B1 and B2, respectively, simulating a split between the now non-interacting stimulus individuals. During the choice phase (average ± SE: 100 ± 25 s), there were no longer any distinct vocal roles. The lack of roles was obtained using a 1.5–10 s stimulus call cadence for each interactant with occasional overlap between them (S2 Table). We considered a flock to make a fusion choice when it took off from its original position after the playback moved and landed within 15 m of either speaker B1 or B2, at which point the choice phase and the trial ended. After a trial had ended, the focal flock’s choice was scored as either a complete fusion, where all individuals in the flock took off and landed in the vicinity of the speaker, or a partial fusion, where only some flock members took off and landed at the speaker. The total size of focal flocks was also noted at this point. Once the focal flock had left the experimental area, a new trial could be attempted.

The design was balanced entirely with regard to vocal role, sex, and identity of the stimulus individuals. Each stimulus individual acted as both leader and follower and was played back from both A1/B1 and A2/B2 throughout the experiment within a field season.

### Data analyses

During each trial, vocal responses from focal flocks were recorded using a Sennheiser MKH 70 directional microphone mounted on a tripod pointing at the respondents and connected to the first channel of a Marantz Professional PMD661 MK II solid-state recorder (sampling rate: 44,100 Hz). We re-recorded all stimulus calls using the same setup to account for any distortion caused by the playback equipment. During playback trials, observers gave comments using a Sennheiser wireless microphone (SK 2000) and receiver (EK 2000) recording to the recorder’s second channel.

All contact call responses from trials and re-recorded stimulus calls were batch extracted using Syrinx 2.6 (John Burt, Pullman, Washington, USA). We counted the number of contact call responses given by focal flocks in the interaction and choice phase of each trial. To determine how well a focal flock matched the playback, we measured the similarity between each contact call given by the focal flock and the re-recorded specific stimulus contact call from the leader and the follower that preceded that particular response. The similarities between responses and re-recorded stimulus contact calls were quantified with the same pairwise SPCC routine used in the stimulus preparation [63]. One male-female trial was excluded from SPCC analyses due to low signal to noise ratio.

We conducted 79 male-male and 111 male-female trials, of which 40 male-male and 40 male-female trials resulted in focal flocks choosing to follow one of the stimulus individuals (42% trial success rate). Successful trials lasted on average 219 ± 13 s (average ± SE) (range: 134–1036 s) and lead to 44 ± 5 contact calls (average ± SE) (range: 2–165 contact calls) from flocks in response to the stimulus calls. The focal flocks used in successful trials consisted of 1–15 individuals (average ± SE: 5 ± 3 individuals).

### Statistics

To test if orange-fronted conures chose to follow leaders significantly more often than expected by chance, we conducted a one-tailed binomial test.

We compared the number of contact calls emitted by focal flocks in each playback phase of trials where they chose the leader or the follower, using generalised linear mixed models (GLMM) with a Poisson distribution and Laplace approximation [64]. As the duration of each trial differed, we accounted for differences in interaction and choice phase length by calculating the number of contact call responses using log (phase duration) as an offset variable, i.e. the response rate. To test whether flock size and fusion type influenced the response rate of focal flocks, we included these as fixed factors in the models. The second-order interaction between flock size and fusion type was not included in the model to allow us to interpret any main effects of fusion type. Location acted as a random factor as we used multiple experimental sites. We tested the male-male and male-female trials separately (S1 Model), as male-female trial models also included the sex of chosen stimulus individuals and the second-order interaction between choice role (follower or leader) and sex as fixed factors. Pearson residuals of response rate models did not differ from a normal distribution and showed no zero-inflation or over-dispersion [64, 65].

We used linear mixed models (LMM) comparing SPCC response-stimulus similarities to test if the role of stimulus individuals affected how well focal flock responses matched the stimulus calls in each playback phase [64]. To compare how well focal flocks matched the stimulus individuals they chose to fuse with, we included choice status (chosen or rejected) and the second-order interaction between choice status and stimulus role (leader or follower) as fixed factors in the models. As in the previous models, the flock size and fusion type were included as fixed factors. As we had multiple observations from the same trials, we accounted for any trial and response order effects by adding these as random factors in the models. Male-male and male-female trials were tested separately (S2 Model). The sex of the stimulus individuals and the second-order interaction between choice status and stimulus sex and between stimulus role and sex were added as fixed factors to male-female trial models. Residuals of SPCC response-stimulus similarities models showed normal distribution and homoscedasticity.

One male-female trial had a considerably longer than average choice phase and could be considered an outlier. However, exclusion of the outlier had no effect on the results, which suggests that the results were robust. We thus included the data point in the final models. Fixed factors of all models were described by calculating parameter estimates and 95% confidence intervals. For discrete factors, we calculated the parameter estimate of the difference between groups and the confidence limit for this difference. We used the least square mean difference (LSMD) with sequential Bonferroni correction of p-values [65] to test specific post hoc pairwise differences in all models. We limited the pairwise comparison of second-order interactions to those where only one fixed factor level differed. For interactions with choice status, we limited the pairwise comparisons of SPCC response-stimulus similarities to those where stimulus individuals were subsequently chosen by focal flocks. All statistics were calculated using proc glimmix and proc mixed in SAS 9.4 (SAS Institute Inc., Cary, North Carolina, USA).

### Ethical statement

According to the Danish Law on animal experimentation LBK no. 474 (May 15^th^, 2014), none of the experiments conducted in this study required a license or special permission as they were nonintrusive without risk of suffering and potential harm to the animals. All activities described were in accordance with the animal welfare in research guidelines from the Department of Experimental Medicine Animal Care and Use Committee at the University of Copenhagen and the European Guidelines for the Treatment of Animals in Behavioural Research and Teaching from the Animal Behaviour Society. The experiments were conducted under the research permits ACG-PI-037-2015 and ACG-PI-027-2016 and approved by the Costa Rican authorities responsible for Área de Concervación Guanacaste.

## Results

Overall, flocks chose to fuse with the leaders (48 trials) of our simulated vocal interactions significantly more often than followers (32 trials) (binomial p = 0.0456). However, we found no significant effect of vocal role on flock choices when analysing male-male and male-female trials separately (S3 Table).

### Response rate

Response rates were significantly affected by the size of focal flocks (Tables 1 and 2).

**Table 1:** Response rate results for male-male trials.

	Interaction phase			Choice phase		
	Estimate (95% CI)	F-value	p-value	Estimate (95% CI)	F-value	p-value
**Choice role**	-0.07 (-0.21, 0.06)	1.19	0.2832	0.36 (0.15, 0.56)	12.35*	0.0013*
**Flock size**	-0.06 (-0.08, -0.03)	17.70*	0.0002*	-0.04 (-0.08, -0.01)	4.99*	0.0322*
**Fusion type**	-0.64 (-0.82, -0.47)	54.39*	< 0.0001*	-0.28 (-0.53, -0.03)	5.16*	0.0295*

Generalised linear mixed model for male-male trials (n = 40) testing the effect of vocal role of the chosen stimulus individual (choice role), flock size and fusion type on the contact call response rate emitted by focal flocks during the interaction and choice phase. For each predictor, significant results are indicated with an asterisk (df_error_ = 34).

**Table 2:** Response rate results for male-female trials.

	Interaction phase			Choice phase		
	Estimate (95% CI)	F-value	p-value	Estimate (95% CI)	F-value	p-value
**Choice role**	-1.59 (-1.81, -1.38)	181.78*	< 0.0001*	-0.54 (-0.79, -0.29)	1.11	0.3010
**Choice sex**	-0.17 (-0.30, -0.04)	44.31*	< 0.0001*	-0.31 (-0.54, -0.09)	12.55*	0.0013*
**Flock size**	-0.03 (-0.05, -0.01)	7.97*	0.0082*	0.05 (0.01, 0.09)	7.94*	0.0084*
**Fusion type**	-1.06 (-1.25, -0.87)	132.22*	< 0.0001*	-0.18 (-0.52, 0.15)	1.23	0.2765
Choice role * Choice sex	1.27 (0.98, 1.56)	81.84*	< 0.0001*	1.28 (0.89, 1.68)	44.12*	< 0.0001*

Generalised linear mixed model for male-female trials (n = 39) testing the effect of vocal role (choice role) and sex (choice sex) of the chosen stimulus individual, flock size and fusion type on the contact call response rate emitted by focal flocks during the interaction and choice phase. For each predictor, significant results are indicated with an asterisk (df_error_ = 31).

**Fig 2:**
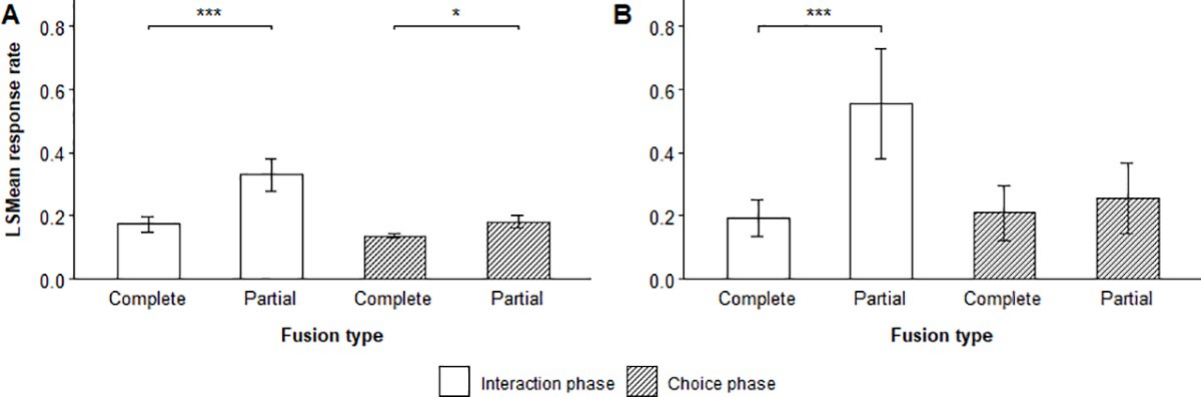
Effect of fusion type on response rate. LSMean (± SE) contact call response rate emitted by focal flocks during the interaction and choice phase of (A) male-male and (B) male-female trials where flocks made partial or complete fusions with a stimulus individual. Significant pairwise comparisons are indicated with asterisks.

Flock size showed a significant negative correlation with the response rate of focal flocks during both the interaction and choice phase of male-male trials (Table 1) and the interaction phase of male-female trials (Table 2), suggesting that larger flocks responded with lower contact call rates than smaller flocks. Conversely, we found a significant positive effect of flock size on the response rate during the choice phase of male-female trials. The response rate also depended on the type of fusion focal flocks made during a trial (Tables 1 and 2). Flocks that made partial fusions with a stimulus individual responded with higher contact call rates during both the interaction and choice phase of male-male trials than flocks where all individuals fused with a stimulus individual (Fig 2).

Male-female trials that resulted in partial fusions also led to higher response rates, but only during the interaction phase (Fig 2).

**Fig 3:**
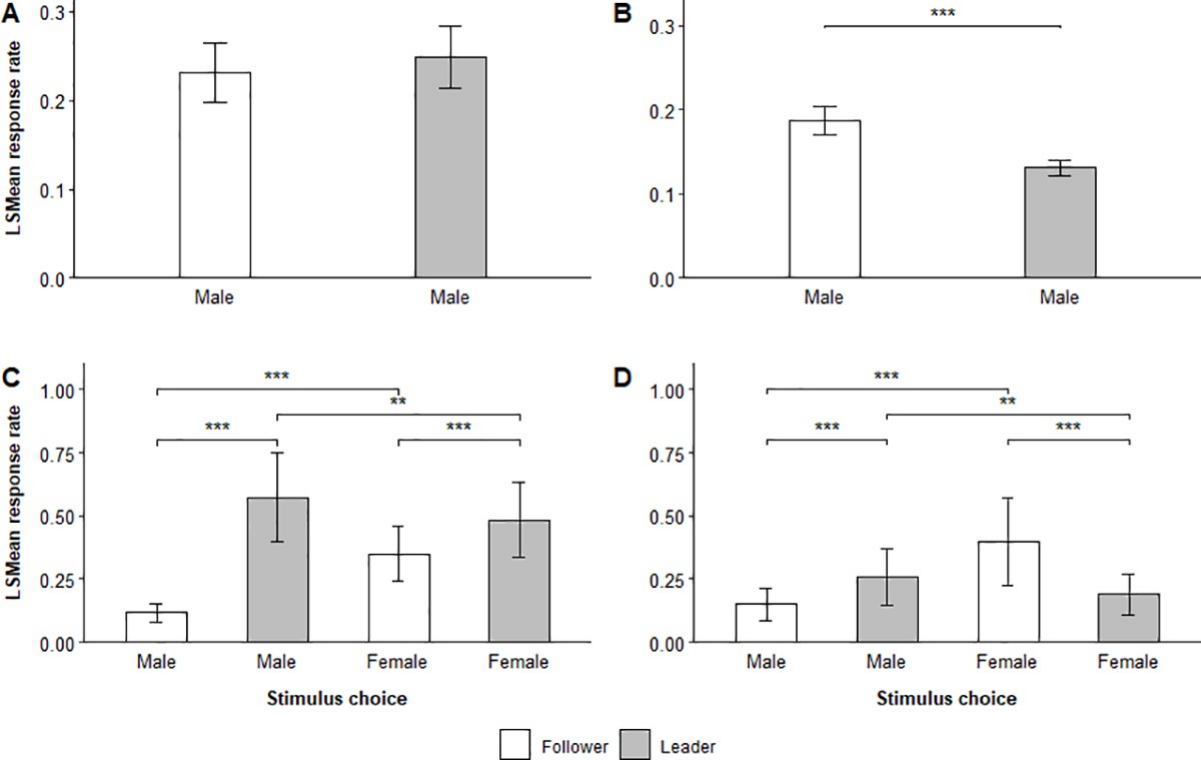
Effect of vocal role and sex on response rate. LSMean (± SE) contact call response rate emitted by focal flocks during the (A) interaction and (B) choice phase of male-male trials where flocks chose the follower or leader, and during the (C) interaction and (D) choice phase of male-female trials where flocks chose a male or female follower or leader. Significant selected pairwise comparisons are indicated with asterisks.

Focal flocks showed considerable variation in how much they responded when choosing the leader and follower of our simulated vocal interactions. In male-male trials, flocks responded with significantly higher contact call rates during the choice phase of trials when they chose the follower compared to trials where they chose the leader (Table 1, Fig 3).

Conversely, flocks responded with higher response rates during the interaction phase of male-female trials where they chose the leader and during both phases of trials where they chose a female stimulus individual. In addition, the interactions between the role and sex of chosen stimulus individuals for male-female trials showed significance in both the interaction and choice phase (Table 2, Fig 3), suggesting that focal flocks responded with different contact call rates to leaders and followers of different sexes. Post hoc analyses of the interaction (S4 Table, S1 Fig) showed that flocks responded with higher contact call rates during both the interaction (LSMD t_31_ = -15.26, p = < 0.0001) and choice phase (LSMD t_31_ = -4.38, p = 0.0001) of trials when they chose a male leader compared to trials where they chose a male follower. Flocks also responded with higher contact call rates in trials where they chose a female leader compared to trials where they chose a female follower, however, only during the interaction phase (LSMD t_31_ = -3.41, p = 0.0018). During the choice phase, flocks responded with significantly higher contact call rates in trials where they chose female followers compared to trials where they chose female leaders (LSMD t_31_ = 4.95, p = < 0.0001).

### Stimulus-response similarity

Several factors affected how well focal flocks matched the stimulus calls during trials. There was a significant effect of flock size on the SPCC similarity between contact call responses and stimulus calls during trials (Tables 3 and 4).

**Table 3:** Stimulus-response similarity results for male trials.

	Interaction phase			Choice phase		
	Estimate (95% CI)	F-value	p-value	Estimate (95% CI)	F-value	p-value
**Choice status**	-0.03 (-0.05, -0.01)	0.30	0.5824	0.04 (0.02, 0.07)	14.50*	0.0002*
**Stimulus role**	-0.03 (-0.05, -0.01)	0.22	0.6373	0.05 (0.02, 0.08)	5.43*	0.0203*
**Flock size**	0.00 (-0.00, 0.01)	0.11	0.7448	0.00 (-0.00, 0.01)	0.00	0.9549
**Fusion type**	0.05 (0.03, 0.07)	24.24*	< 0.0001*	0.06 (0.02, 0.10)	8.18*	0.0045*
Choice status * Stimulus role	0.05 (0.02, 0.09)	8.06*	0.0046*	-0.13 (-0.18. -0.07)	22.64*	< 0.0001*

Linear mixed model analysing the spectrographic cross-correlation similarity between stimulus calls and focal flock responses for male-male trials (n = 33). The model tests the effect of choice status and role of stimuli individuals, focal flock size and fusion type for the interaction and choice phase. For each predictor and the interaction effect, significant results are indicated with an asterisk (interaction phase df_error_ = 737, choice phase df_error_ = 353).

**Table 4:** Stimulus-response similarity results for male-female trials.

	Interaction phase			Choice phase		
	Estimate (95% CI)	F-value	p-value	Estimate (95% CI)	F-value	p-value
**Choice status**	0.40 (0.36, 0.44)	1.07	0.3015	-0.00 (-0.04, 0.03)	5.34*	0.0212*
**Stimulus role**	0.38 (0.32, 0.44)	0.77	0.3810	0.01 (-0.02, 0.05)	5.51*	0.0193*
**Stimulus sex**	0.34 (0.28, 0.40)	2.99	0.0839	0.01 (-0.02, 0.04)	1.88	0.1710
**Flock size**	-0.00 (-0.01, -0.00)	4.30*	0.0383*	0.01 (0.01, 0.01)	24.63*	< 0.0001*
**Fusion type**	-0.07 (-0.12, -0.03)	9.01*	0.0027*	0.05 (0.01, 0.08)	7.52*	0.0063*
Choice status * Stimulus role	-0.45 (-0.52, -0.37)	132.24*	< 0.0001*	-0.02 (-0.06, 0.03)	0.62	0.4328
Choice status * Stimulus sex	-0.34 (-0.41, -0.27)	91.88*	< 0.0001*	-0.00 (-0.05, 0.04)	0.03	0.8689
Stimulus role * Stimulus sex	-0.33 (-0.40, -0.26)	83.35*	< 0.0001*	-0.03 (-0.07, 0.01)	1.89	0.1693

Linear mixed model analysing the spectrographic cross-correlation similarity between stimulus calls and focal flock responses for male-female trials (n = 37). The model tests the effect of choice status, role and sex of stimulus individuals as well as focal flock size and fusion type for the interaction and choice phase. For each predictor and the interaction effect, significant results are indicated with an asterisk (interaction phase df_error_ = 1167, choice phase df_error_ = 613).

Flock size showed a significant negative correlation with SPCC stimulus-response similarity during the interaction phase of male-female trials (Table 3), with smaller flocks responding with better matches of stimulus calls than larger flocks. Conversely, we found a significant positive effect of flock size on the response-stimulus similarity during the choice phase of male-female trials. There was no effect of flock size in male-male trials (Table 4).

**Fig 4:**
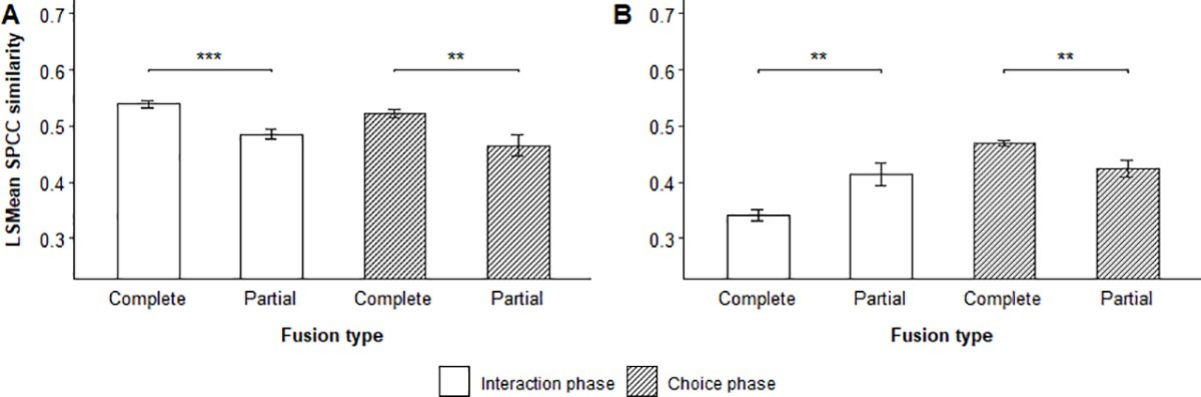
Effect of fusion type on stimulus-response similarity. LSMean (± SE) spectrographic cross-correlation (SPCC) similarity between stimulus calls and focal flock responses emitted during the interaction and choice phase of (A) male-male and (B) male-female trials that resulted in partial and complete fusions with a stimulus individual. Significant pairwise comparisons are indicated with asterisks.

The SPCC similarity between contact call responses and stimulus calls also depended on the type of fusion focal flocks made during trials (Tables 3 and 4). Focal flocks that made complete fusions with a stimulus individual responded with contact calls that better matched the stimulus calls during both phases of male-male trials and the choice phase of male-female trials than flocks that made partial fusion (Fig 4).

Inversely, responses emitted during the interaction phase of male-female trials better matched the stimulus calls in trials where flocks made partial fusions with a stimulus individual (Fig 4).

**Fig 5:**
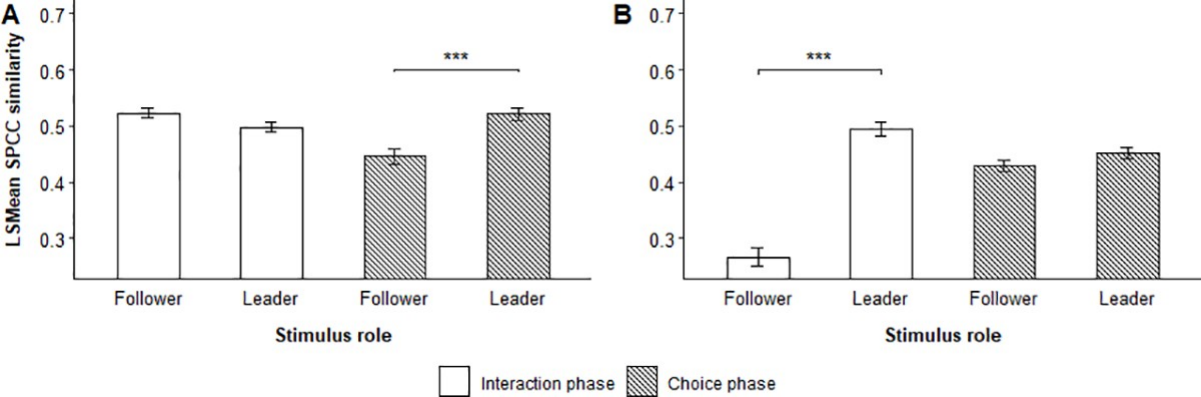
Effect of chosen vocal role on stimulus-response similarity. LSMean (± SE) spectrographic cross-correlation (SPCC) similarity between stimulus calls and focal flock responses emitted during the interaction and choice phase of (A) male-male trials and (B) male-female trials. The figure shows the difference between leaders and followers that were chosen by focal flocks. Significant selected pairwise comparisons are indicated with asterisks.

Focal flocks showed considerable variation in how well they matched the stimulus calls from leaders and followers when they chose to follow them, as indicated by the significant interaction between stimulus role and choice status (Tables 3 and 4) in the interaction and choice phase of male-male trials, and the interaction phase of male-female trials (Fig 5, S5 and S6 Tables, S2 and S3 Figs).

Overall, focal flocks responded with contact calls that matched stimulus calls from leaders better than those from followers during the choice phase of both male-male and male-female trials. Stimulus individuals acting as leaders were generally matched the best when they were subsequently chosen by focal flocks as indicated by the stimulus-response similarity during the choice phase of male-male trials (LSMD t_353_ = 3.03, p = 0.0026) and interaction phase of male-female trials (LSMD t_1167_ = 11.36, p = < 0.0001). Consequently, we found an overall effect of choice status on the SPCC stimulus-response similarity during the choice phase of male-male and male-female trials.

**Fig 6:**
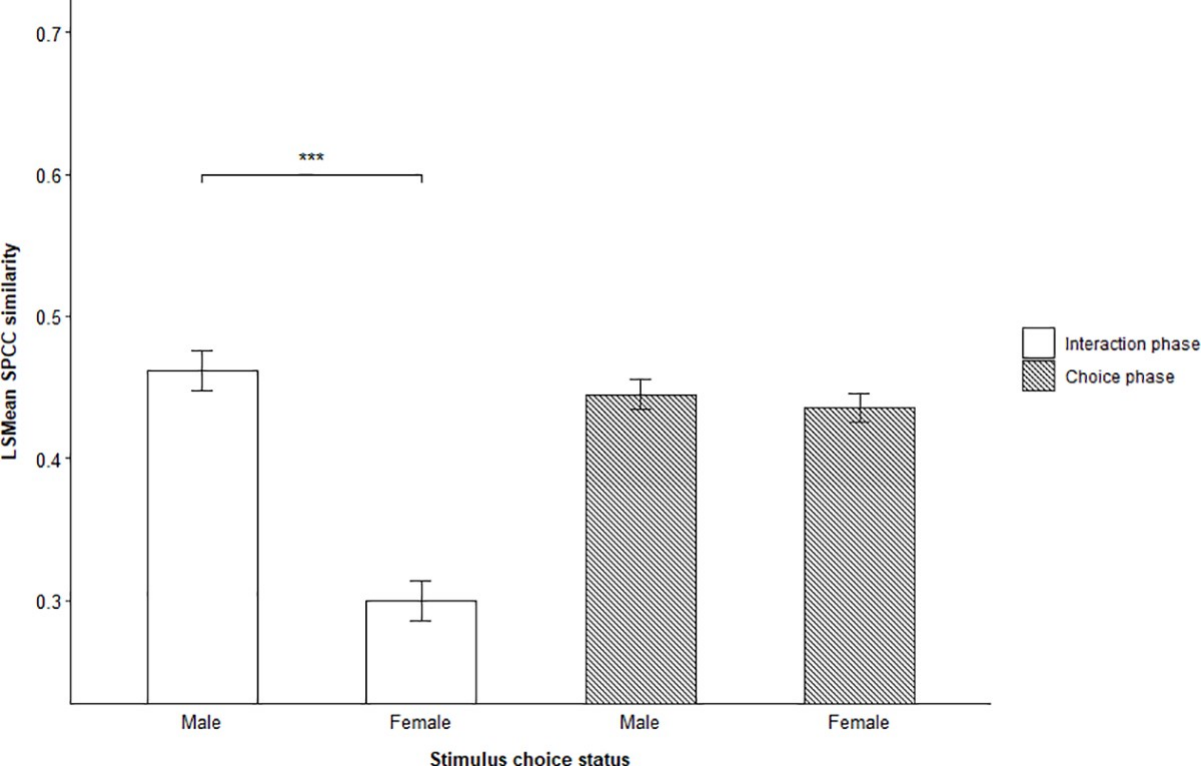
Effect of chosen sex on stimulus-response similarity. LSMean (± SE) spectrographic cross-correlation (SPCC) similarity between stimulus calls and focal flock responses emitted during the interaction and choice phase of male-female trials. The figure shows the difference between males and females that were chosen by focal flocks. Significant selected pairwise comparisons are indicated with asterisks.

The results showed similar patterns for flock responses to chosen stimulus individuals of different sexes (Table 4, Fig 6).

**Fig 7:**
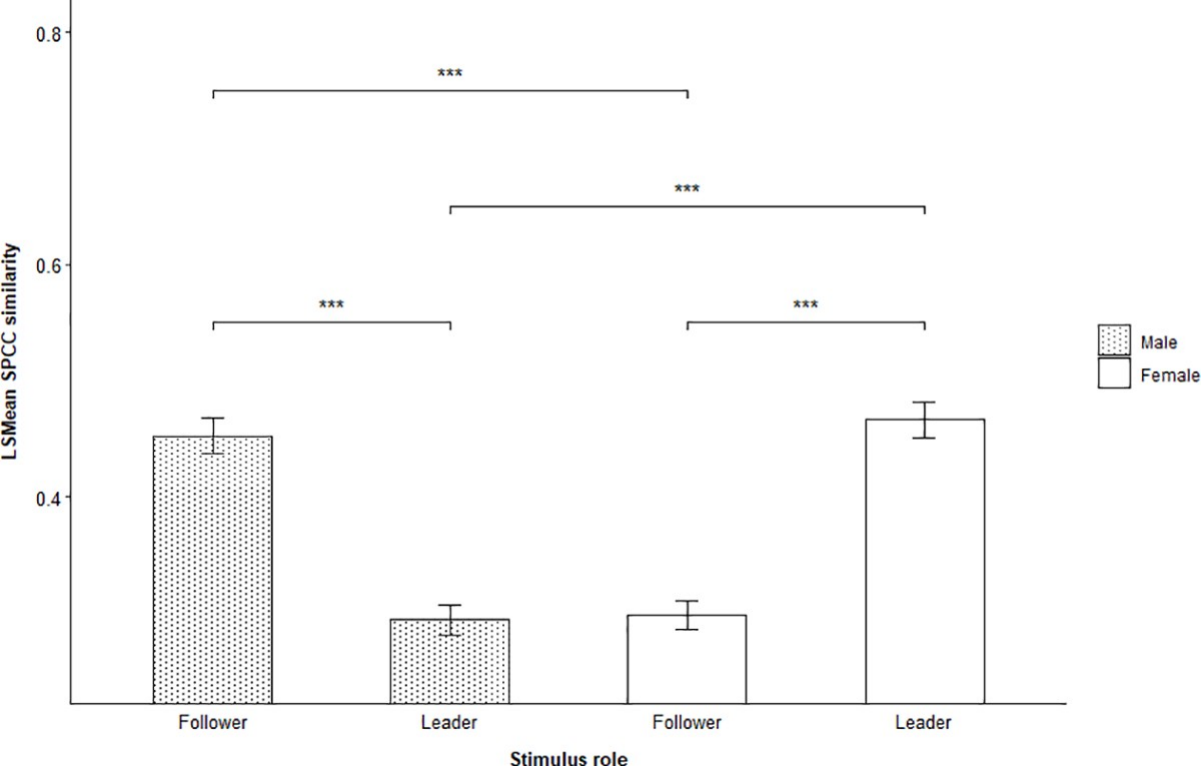
Effect of vocal role and sex on stimulus-response similarity. LSMean (± SE) spectrographic cross-correlation (SPCC) similarity between stimulus calls and focal flock responses emitted during the interaction phase of male-female trials. The figure shows the difference between leaders and followers of both sexes. Significant selected pairwise comparisons are indicated with asterisks.

Male stimulus individuals were matched significantly better during the interaction phase of male-female trials when focal flocks chose to follow them compared to females (LSMD t_1167_ = -8.71, p = < 0.0001) (S7 Table, S4 Fig). When disregarding if focal flocks chose stimulus individuals, we found an overall interaction between the role and sex of stimulus individuals on the SPCC stimulus-response similarity during male-female trials (Table 4, Fig 7), suggesting that focal flocks showed considerable variation in how well they matched stimulus calls from leaders and followers of both sexes.

Post hoc pairwise comparisons showed that stimulus calls from female leaders were matched better than calls from female followers (LSMD t_1167_ = -8.98, p = < 0.0001) and male leaders (LSMD t_1167_ = 9.25, p = < 0.0001), whereas flock responses to male followers were more similar to stimulus calls than responses to male leaders (LSMD t_1167_ = 8.46, p = < 0.0001) and female followers (LSMD t_1167_ = -8.27, p = < 0.0001) (S8 Table, S5 Fig). However, this pattern was only found for contact calls emitted by flocks during the interaction phase.

## Discussion

Using a multi-speaker playback experiment with wild orange-fronted conures allowed us to test novel aspects of contact call matching. Although we cannot exclude the use of other information, our findings are consistent with the hypothesis that orange-fronted conure flocks utilise social information acquired by eavesdropping on vocal interactions to infer the relative quality of unfamiliar individuals. In our playback experiment, we simulated a contact call interaction between two individuals in which a follower matched a leader. When playing these interactions to wild flocks of orange-fronted conures, focal flocks showed differentiated responses towards leaders and followers, suggesting that they extracted sequential information about the vocal pattern embedded in the interaction. Furthermore, when given a choice between interactants, focal flocks showed different preferences for following leaders and followers. As this information can only be derived by listening to both interactants simultaneously, it is reasonable to suggest that focal flocks perceived the playback stimuli as two distinct individuals. Subsequently, inferring the relative quality of interactants based on their vocal matching behaviour in the simulated interaction. Yet, the way individuals responded to this social information substantially differed between flocks.

We found a general negative relationship between the response rate and flock size in both male-male and male-female trials (Tables 1 and 2). Although we could not explicitly test this, our results suggest that not all members of orange-fronted conure flocks participate simultaneously in contact call interactions prior to fusion. Our results indicate that there may be asymmetries in leadership roles within flocks, a notion suggested by [34] and supported by the rapid and directed manner with which conures move between foraging sites [41]. Hence, the exchange of contact calls could be some form of negotiation between a few individuals, e.g. to determine the subsequent leadership should the two flocks fuse or establishing a hierarchy [37]. Such an effect would be more prominent in larger flocks and could explain the negative relations between contact call rate and flock size observed in our experiment. Instead, ‘silent’ individuals could acquire information about potential recruits by eavesdropping on their interaction with other flock members [66]. Individuals may differ in their tendency to interact with others directly and can consistently act as vocal interactants or eavesdroppers as a function of their personality [67], social dominance [68–70] and demography [71]. Alternatively, variation in an individual’s information status and motivation [72] may counteract consistency in the information acquisition and favour opportunistic use of conspecific information from flock members [73]. For example, food-deprived fish have been shown to take the front position in shoals, where they have a more decisive influence on movement direction and increased feeding rates [74]. Furthermore, females in energetically demanding reproductive states often assume the leading positions in white-handed gibbons (*Hylobates lar*) [75].

Not all individuals in focal flocks chose to follow a stimulus individual, and nine male-male and seven male-female trials resulted in partial fusions. Most models dealing with animal group decision-making assume that individuals only receive grouping benefits if all members adhere to the same decision and stay within the group [76–78]. However, differences in several ecological and social factors are likely to create heterogeneous decision preferences between members in groups [76, 79–81]. Consequently, a consensus is more difficult to achieve where individuals end up with unequal pay-offs from a decision due to uneven needs [82]. Several hypotheses have been proposed to explain fission-fusion dynamics in animals [83, 84]. Where the costs of maintaining group cohesion become too high, a group can temporarily split, offering a strategy to balance the costs and benefits of association [84]. For example, studies on communal roost decisions in Bechstein’s bat (*Myotis bechsteinii*) revealed that the decision rule used depended on how strongly individual interests diverged between colony members [85, 86]. Similarly, chacma baboon (*Papio ursius*) groups split when individuals had weak social links to the initiator of group foraging decisions [70]. We found that orange-fronted conure flocks responded with higher contact call rates during trials that ended in partial fusions (Fig 2). Such an increase in response rate could be explained by increased arousal during decision disagreements in flocks [87] or more individuals trying to influence the decision outcome [80]. In contrasts, flocks matched the stimulus calls better in trials where all individuals made the same decision (Fig 4). When combined, these results suggest that recruitment may sometimes result in conflicts of interest between flock members. Fissions may thus allow orange-fronted conures to avoid consensus decisions that are not in their favour without foregoing the flocking benefits that arise from collective behaviour [46].

Our statistical models showed several significant interactions between the performance role and sex of stimulus individuals, suggesting that focal flocks responded differently to leaders and followers of different sexes. Overall, focal flocks responded with higher response rates during trials where they decided to follow male and female leaders compared to when they chose to follow followers (Fig 3). Furthermore, we found a significant effect of sex on the SPCC stimulus-response similarity during trials, with chosen males being matched significantly closer than chosen females (Table 4). When combined, these results speak in favour of focal flocks responding to preferably male leaders. The reason for responding more strongly to males may be that males are higher in rank than females [88, 89] or produce contact calls more often [37]. However, this needs to be tested further. Therefore, a suitable extension of this study would be to test for effects of the sex of interactants on the eavesdropping behaviour of orange-fronted conures in a more controlled environment, such as a temporary capture setting [e.g. 37].

Vocal role of the interactant chosen by flocks represent one of the most influential determinants of response intensity and matching quality. Contact calls from chosen leaders were generally matched the closest by flocks (Fig 4). More importantly, we found a flock preference for following leaders over followers across trials. Following individuals being vocally matched could be the result of affiliative decision copying [52]. Preferences for specific social partners are likely to emerge in dynamic social systems where individuals have different value and may present individuals with various potential benefits depending on the social context [90]. Orange-fronted conures forage predominantly on fruit, flowers and seeds with short seasonal resource availability [42, 91]. Nancite fruits (*Byrsonima crassifolia*) represent an essential ephemeral food resource for orange-fronted conures during the early wet season [41, 92]. Thus, foraging resources may be costly, challenging, and, in some cases, unfeasible for individual orange-fronted conures to monopolise. Given these challenges, individuals without sufficient information would benefit by following conspecifics with greater knowledge of the quality of previously visited nancite sites [6, 93]. In particular, social network position plays a critical role in determining who acquire novel information and from whom [69, 70, 94]. Individuals central in these networks are more likely than noncentral individuals to learn novel information from their conspecifics [95]. Being socially connected to successful individuals would provide immediate or future opportunities to observe and acquire information from them. For example, in golden shiner fish (*Notemigonus crysoleucas*) [96] and common ravens (*Corvus corax*) [97], individuals with superior information can lead groups to high-quality resources, and broad-winged hawks (*Buteo platypterus*) follow experienced elders during migration [98]. Thus, central individuals are likely to become valuable social partners and receive more social connections from the conspecifics who observe them [69, 99, 100]. Following interaction leaders may allow uninformed orange-fronted conures to optimise their foraging decisions as being matched could predict that individuals are more central and more influential as information spreaders [78, 101]. For example, brown-throated conures (*Eupsittula pertinax*) display contact calling behaviours that may function as a means of active information sharing [49]. Individuals in flocks flying past a foraging flock were more likely to land if a member of the foraging flock called. However, foraging flocks did not call to all flying flocks, which indicates that individuals may selectively share information [49]. The cryptic foraging style of many parrots [92] likely makes scrounging foraging information from observations more difficult for flying flocks. As a result, reciprocal sharing of foraging information may thus be essential for locating suitable foraging resources.

In summary, our findings illustrate the complexity of vocal interactions in orange-fronted conures, highlighting that dyadic interactions do not represent an appropriate framework to understand the function of contact call matching in the flock decisions of parrots. While orange-fronted conures frequently engage in contact call matching during vocal interactions with conspecifics [34], we illustrate that vocal matching also has implications for potential third parties. These birds are selective with how they respond to interactants, indicating that contact call matching plays an essential role in facilitating the formation and maintenance of affiliative social interactions. Using a large-scale experimental design with wild flocks, we demonstrated that vocal roles are salient to orange-fronted conures, a previously missing key to understanding the function of affiliative vocal matching in parrots. While several questions remain, our experimental design demonstrated the feasibility of conducting choice experiments with individuals in the wild, thereby expanding the possibilities of future research in animals’ social decisions.

## Supporting information

S1 Table Stimulus setup used in the interaction phase of playbacks. The table shows stimulus call cadence measured from the start of each call to the start of the next (in seconds) for leader and follower and the latency between the corresponding leader and follower calls. The average spectrographic cross-correlation (SPCC) similarity is shown for each leader-follower stimulus call pair with low, intermediate, and high similarity pairs indicated by the greyscale.
(DOCX)

S2 Table Stimulus setup used in the choice phase of playbacks. The table shows the stimulus call cadence measured from the start of each call to the start of the next (in seconds) for the leader and follower. The average spectrographic cross-correlation (SPCC) similarity is given for each leader-follower stimulus call pair.
(DOCX)

S3 Table Binomial exact test results. Test results for the choice of focal flocks to follow interaction leaders in male-male trials (n = 40), male-female trials (n = 40), and when both trial types were combined (n = 80). Significant results are indicated with an asterisk.
(DOCX)

S4 Table Post hoc pairwise differences (Choice role * Choice sex) in contact call rate of male-female trials. LSMean differences in the number of contact calls given by focal flocks during the interaction and choice phase of male-female trials (n = 39). The table shows each pairwise comparison of interactions between the role and sex of stimulus individuals that focal flocks chose to follow. Significant results are indicated with an asterisk.
(DOCX)

S5 Table Post hoc pairwise differences (Choice status * Stimulus role) in stimulus-response similarity of male-male trials. LSMean differences in the spectrographic cross-correlation similarity between focal flock responses and stimulus calls in the interaction (n = 31) and choice phase (n = 25) of male-male trials. The table shows each pairwise comparison of interactions between the choice status (chosen or not chosen by flocks) and the role of stimulus individuals. Significant results are indicated with an asterisk.
(DOCX)

S6 Table Post hoc pairwise differences (Choice status * Stimulus role) in stimulus-response similarity of male-female trials. LSMean differences in the spectrographic cross-correlation similarity between focal flock responses and stimulus calls in the interaction phase (n = 34) of male-female trials. The table shows each pairwise comparison of interactions between the choice status (chosen or not chosen by flocks) and the role of stimulus individuals stimulus role. Significant results are indicated with an asterisk.
(DOCX)

S7 Table Post hoc pairwise differences (Choice status * Stimulus sex) in stimulus-response similarity of male-female trials. LSMean differences in the spectrographic cross-correlation similarity between focal flock responses and stimulus calls in the interaction phase (n = 34) of male-female trials. The table shows each pairwise comparison of interactions between the choice status (chosen or not chosen by flocks) and sex of stimulus individuals. Significant results are indicated with an asterisk.
(DOCX)

S8 Table Post hoc pairwise differences (Stimulus role * Stimulus sex) in stimulus-response similarity of male-female trials. LSMean differences in the spectrographic cross-correlation similarity between focal flock responses and stimulus calls in the interaction phase (n = 34) of male-female trials. The table shows each pairwise comparison of interactions between the role and sex of stimulus individuals. Significant results are indicated with an asterisk.
(DOCX)

S1 Fig Forest plot of post hoc pairwise differences (Choice role * Choice sex) in contact call rate of male-female trials. Model estimates and 95% confidence intervals of the LSMean difference in the number of contact calls given by focal flocks during the interaction and choice phase of male-female trials. The figure shows each pairwise comparison of interactions between role (F = follower and L = leader) and sex (M = male and F = female) of stimulus individuals that focal flocks chose to follow.
(TIF)

S2 Fig Forest plot of post hoc pairwise differences (Choice status * Stimulus role) in stimulus-response similarity of male-male trials. Model estimates and 95% confidence intervals of the LSMean difference in the spectrographic cross-correlation similarity between flock responses and stimulus calls in the interaction and choice phase of male-male trials. The figure shows each pairwise comparison of interactions between the choice status (C = chosen and R = not chosen) and role (L = leader and F = follower) of stimulus individuals.
(TIF)

S3 Fig Forest plot of post hoc pairwise differences (Choice status * Stimulus role) in stimulus-response similarity of male-female trials. Model estimates and 95% confidence intervals of the LSMean difference in the spectrographic cross-correlation similarity between flock responses and stimulus calls in the interaction phase of male-female trials. The figure shows each pairwise comparison of interactions between the choice status (C = chosen and R = not chosen) and role (L = leader and F = follower) of stimulus individuals.
(TIF)

S4 Fig Forest plot of post hoc pairwise differences (Choice status * Stimulus sex) in stimulus-response similarity of male-female trials. Model estimates and 95% confidence intervals of the LSMean difference in the spectrographic cross-correlation similarity between flock responses and stimulus calls in the interaction phase of male-female trials. The figure shows each pairwise comparison of interactions between the choice status (C = chosen and R = not chosen) and sex (M = male and F = female) of stimulus individuals.
(TIF)

S5 Fig Forest plot of post hoc pairwise differences (Stimulus role * Stimulus sex) in stimulus-response similarity of male-female trials. Model estimates and 95% confidence intervals of the LSMean difference in the spectrographic cross-correlation similarity between flock responses and stimulus calls in the interaction phase of male-female trials. The figure shows each pairwise comparison of interactions between the role (F = Follower and L = leader) and sex (M = male and F = female) of stimulus individuals.
(TIF)

S1 Model Generalised Linear Mixed Models (GLMM). The model for number of contact calls (response rate) that focal flocks emitted during (A) male-male and (B) male-female trials. Lower case letters show the fixed factors with second-order interactions shown as multiplications indicated with an asterisk. Capital letters correspond to any random factors added to the model.
(DOCX)

S2 Model Linear Mixed Models (LMM). The model for spectrographic cross-correlation similarity between contact call responses from focal flocks and stimulus calls (response-playback similarity) in (A) male-male and (B) male-female trials. Lower case letters show the fixed factors with second-order interactions shown as multiplications indicated with an asterisk. Capital letters correspond to any random factors added to the model.
(DOCX)
