# Supplementary material for: Follow the leader? Orange-fronted conures eavesdrop on conspecific vocal performance and utilise it in social decisions
Source: PLoS One. 2021 Jun 9;16(6):e0252374. doi: 10.1371/journal.pone.0252374 (PMC8189466; doi:10.1371/journal.pone.0252374)
Supplement: S1 Table — The table shows stimulus call cadence measured from the start of each call to the start of the next (in seconds) for leader and follower and the latency between the corresponding leader and follower calls. The average spectrographic cross-correlation (SPCC) similarity is shown for each leader-follower stimulus call pair with low, intermediate, and high similarity pairs indicated by the greyscale. (DOCX) [file pone.0252374.s001.docx]

|  | **Stimulus call sequence** | | | | | | | | | | | | | | |
| --- | --- | --- | --- | --- | --- | --- | --- | --- | --- | --- | --- | --- | --- | --- | --- |
|  | **1** | **2** | **3** | **4** | **5** | **6** | **7** | **8** | **9** | **10** | **11** | **12** | **13** | **14** | **15** |
| **Leader cadence** | - | 9 | 10 | 8 | 10 | 8 | 6 | 7 | 8 | 6 | 8 | 8 | 7 | 10 | 7 |
| **Follower cadence** | - | 9 | 10.5 | 9.5 | 9 | 7 | 7 | 6 | 8 | 6 | 8 | 9 | 6 | 11 | 7 |
| **Latency** | 1 | 1 | 1.5 | 3 | 2 | 1 | 2 | 1 | 1 | 1 | 1 | 2 | 1 | 2 | 2 |
| **SPCC similarity** | 0.45 | 0.46 | 0.48 | 0.49 | 0.50 | 0.53 | 0.56 | 0.59 | 0.62 | 0.65 | 0.75 | 0.77 | 0.79 | 0.79 | 0.79 |
