# Supplementary material for: Follow the leader? Orange-fronted conures eavesdrop on conspecific vocal performance and utilise it in social decisions
Source: PLoS One. 2021 Jun 9;16(6):e0252374. doi: 10.1371/journal.pone.0252374 (PMC8189466; doi:10.1371/journal.pone.0252374)
Supplement: S2 Table — The table shows the stimulus call cadence measured from the start of each call to the start of the next (in seconds) for the leader and follower. The average spectrographic cross-correlation (SPCC) similarity is given for each leader-follower stimulus call pair. (DOCX) [file pone.0252374.s002.docx]

|  | **Stimuli call sequence** | | | | | | | | | | | |
| --- | --- | --- | --- | --- | --- | --- | --- | --- | --- | --- | --- | --- |
|  | **1** | **2** | **3** | **4** | **5** | **6** | **7** | **8** | **9** | **10** | **11** | **12** |
| **Leader cadence** | 17 | 8 | 9 | 10 | 6 | 1.5 | 8 | 7 | 1.5 | 9 | 8 | 7 |
| **Follower cadence** | 16.5 | 8 | 1.5 | 10 | 6 | 7 | 8 | 7 | 10 | 1.5 | 8 | 7 |
| **SPCC similarity** | 0.77 | 0.78 | 0.78 | 0.78 | 0.78 | 0.78 | 0.77 | 0.78 | 0.78 | 0.78 | 0.78 | 0.78 |
