# Supplementary material for: Follow the leader? Orange-fronted conures eavesdrop on conspecific vocal performance and utilise it in social decisions
Source: PLoS One. 2021 Jun 9;16(6):e0252374. doi: 10.1371/journal.pone.0252374 (PMC8189466; doi:10.1371/journal.pone.0252374)
Supplement: S3 Table — Test results for the choice of focal flocks to follow interaction leaders in male-male trials (n = 40), male-female trials (n = 40), and when both trial types were combined (n = 80). Significant results are indicated with an asterisk. (DOCX) [file pone.0252374.s003.docx]

|  | **Male-male trials** | **Male-female trials** | **All trials** |
| --- | --- | --- | --- |
| **Leader frequency** | 24 | 24 | 48 |
| **Leader proportion** | 0.6 | 0.6 | 0.6 |
| **95% lower CL (exact)** | 0.4333 | 0.4333 | 0.4844 |
| **95% upper CL (exact)** | 0.7514 | 0.7514 | 0.7080 |
| **p (one-tailed)** | 0.1341 | 0.1341 | 0.0465* |
