# Supplementary material for: Follow the leader? Orange-fronted conures eavesdrop on conspecific vocal performance and utilise it in social decisions
Source: PLoS One. 2021 Jun 9;16(6):e0252374. doi: 10.1371/journal.pone.0252374 (PMC8189466; doi:10.1371/journal.pone.0252374)
Supplement: S4 Table — LSMean differences in the number of contact calls given by focal flocks during the interaction and choice phase of male-female trials (n = 39). The table shows each pairwise comparison of interactions between the role and sex of stimulus individuals that focal flocks chose to follow. Significant results are indicated with an asterisk. (DOCX) [file pone.0252374.s004.docx]

|  |  | **Interaction phase** | | | | **Choice phase** | | | |
| --- | --- | --- | --- | --- | --- | --- | --- | --- | --- |
| **Choice role ***  **Choice sex** | **Choice role ***  **Choice sex** | **Estimate**  **(95% CI)** | **t-value** | **p-value** | **Bonferroni**  **p-value** | **Estimate**  **(95% CI)** | **t-value** | **p-value** | **Bonferroni**  **p-value** |
| Follower*  Female | Follower*  Male | 1.10  (0.85, 1.35) | 8.87 | < 0.0001* | < 0.0006* | 0.97  (0.66, 1.29) | 6.31 | < 0.0001* | < 0.0006* |
| Follower*  Female | Leader*  Female | -0.32  (-0.52, -0.13) | -3.41 | 0.0018* | 0.0036* | 0.75  (0.44, 1.05) | 4.95 | < 0.0001* | < 0.0006* |
| Follower*  Female | Leader*  Male | -0.49  (-0.67, -0.32) | -5.70 | < 0.0001* | < 0.0006* | 0.43  (0.13, 0.74) | 2.89 | 0.0070* | 0.021* |
| Follower*  Male | Leader*  Female | -1.42  (-1.65, -1.20) | -12.82 | < 0.0001* | < 0.0006* | -0.23  (-0.47, 0.02) | -1.91 | 0.0664 | 0.0654 |
| Follower*  Male | Leader*  Male | -1.60  (-1.81, -1.38) | -15.26 | < 0.0001* | < 0.0006* | -0.54  (-0.79, -0.29) | -4.38 | 0.0001* | 0.0006* |
| Leader*  Female | Leader*  Male | -0.17  (-0.30, -0.04) | -2.62 | 0.0135* | 0.0135* | -0.31  (-0.54, -0.09) | -2.83 | 0.0081* | 0.0162* |
