# Supplementary material for: Follow the leader? Orange-fronted conures eavesdrop on conspecific vocal performance and utilise it in social decisions
Source: PLoS One. 2021 Jun 9;16(6):e0252374. doi: 10.1371/journal.pone.0252374 (PMC8189466; doi:10.1371/journal.pone.0252374)
Supplement: S5 Table — LSMean differences in the spectrographic cross-correlation similarity between focal flock responses and stimulus calls in the interaction (n = 31) and choice phase (n = 25) of male-male trials. The table shows each pairwise comparison of interactions between the choice status (chosen or not chosen by flocks) and the role of stimulus individuals. Significant results are indicated with an asterisk. (DOCX) [file pone.0252374.s005.docx]

|  | |  | | **Interaction phase** | | | | | **Choice phase** | | | |
| --- | --- | --- | --- | --- | --- | --- | --- | --- | --- | --- | --- | --- |
| **Choice status ***  **Stimulus role** | **Choice status ***  **Stimulus role** | | **Estimate**  **(95% CI)** | | **t-value** | **p-value** | **Bonferroni**  **p-value** | **Estimate**  **(05% CI)** | | **t-value** | **p-value** | **Bonferroni**  **p-value** |
| Chosen*  Follower | Chosen*  Leader | | 0.02  (0.00, 0.05) | | 2.26 | 0.0244* | 0.0976 | -0.08  (-0.10, -0.05) | | -5.28 | < 0.0001* | < 0.0005* |
| Chosen*  Follower | Rejected*  Follower | | 0.02  (0.00, 0.05) | | 2.22 | 0.0269* | 0.0807 | -0.08  (-0.11, -0.06) | | -5.82 | < 0.0001* | < 0.0006* |
| Chosen*  Follower | Rejected*  Leader | | -0.01  (-0.02, 0.01) | | -0.69 | 0.4882 | 0.9764 | -0.03  (-0.05, -0.02) | | -3.88 | 0.0001* | 0.0004* |
| Chosen*  Leader | Rejected*  Follower | | -0.00  (-0.01, 0.01) | | -0.06 | 0.9538 | 0.9538 | -0.01  (-0.02, 0.00) | | -1.21 | 0.2285 | 0.2285 |
| Chosen*  Leader | Rejected*  Leader | | -0.03  (-0.05, -0.01) | | -2.75 | 0.0061* | 0.0366* | 0.04  (0.02, 0.07) | | 3.03 | 0.0026* | 0.0052* |
| Rejected*  Follower | Rejected*  Leader | | -0.03  (-0.05, -0.01) | | -2.71 | 0.0068* | 0.034* | 0.05  (0.02, 0.08) | | 3.57 | 0.0004* | 0.0012* |
