# Supplementary material for: Follow the leader? Orange-fronted conures eavesdrop on conspecific vocal performance and utilise it in social decisions
Source: PLoS One. 2021 Jun 9;16(6):e0252374. doi: 10.1371/journal.pone.0252374 (PMC8189466; doi:10.1371/journal.pone.0252374)
Supplement: S8 Table — LSMean differences in the spectrographic cross-correlation similarity between focal flock responses and stimulus calls in the interaction phase (n = 34) of male-female trials. The table shows each pairwise comparison of interactions between the role and sex of stimulus individuals. Significant results are indicated with an asterisk. (DOCX) [file pone.0252374.s008.docx]

|  |  | **Interaction phase** | | | |
| --- | --- | --- | --- | --- | --- |
| **Stimulus role ***  **Stimulus sex** | **Stimulus role ***  **Stimulus sex** | **Estimate**  **(95% CI)** | **t-value** | **p-value** | **Bonferroni**  **p-value** |
| Follower*  Female | Follower*  Male | -0.15  (-0.19, -0.12) | -8.27 | < 0.0001* | < 0.0003* |
| Follower*  Female | Leader*  Female | -0.17  (-0.20, -0.13) | -8.98 | < 0.0001* | < 0.0005* |
| Follower*  Female | Leader*  Male | 0.00  (-0.01, 0.02) | 0.65 | 0.5164 | 0.5164 |
| Follower*  Male | Leader*  Female | -0.01  (-0.03, 0.00) | -1.62 | 0.1064 | 0.2128 |
| Follower*  Male | Leader*  Male | 0.16  (0.12, 0.20) | 8.46 | < 0.0001* | < 0.0004* |
| Leader*  Female | Leader*  Male | 0.17  (0.14, 0.21) | 9.25 | < 0.0001* | < 0.0006* |
