# Supplementary figures and images for: Follow the leader? Orange-fronted conures eavesdrop on conspecific vocal performance and utilise it in social decisions
Source: PLoS One. 2021 Jun 9;16(6):e0252374. doi: 10.1371/journal.pone.0252374 (PMC8189466; doi:10.1371/journal.pone.0252374)

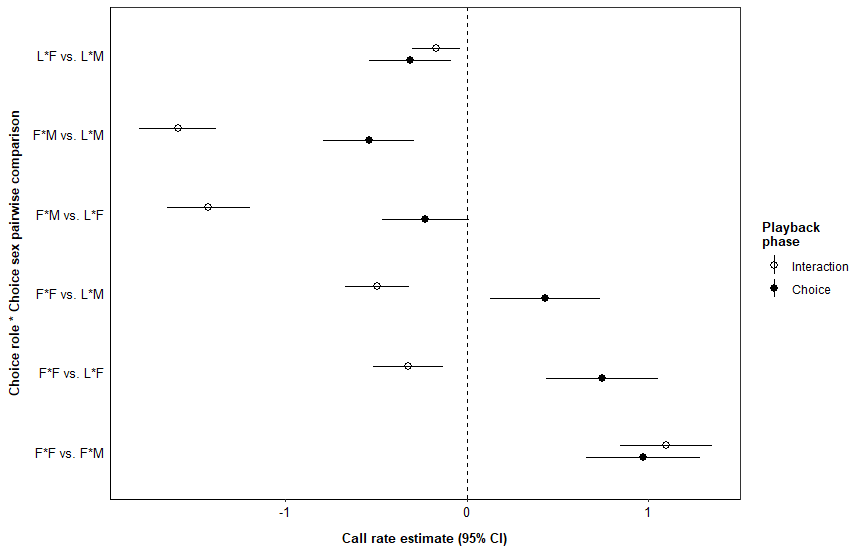

Supplement: S1 Fig — Model estimates and 95% confidence intervals of the LSMean difference in the number of contact calls given by focal flocks during the interaction and choice phase of male-female trials. The figure shows each pairwise comparison of interactions between role (F = follower and L = leader) and sex (M = male and F = female) of stimulus individuals that focal flocks chose to follow. (TIF) [file pone.0252374.s009.tif]

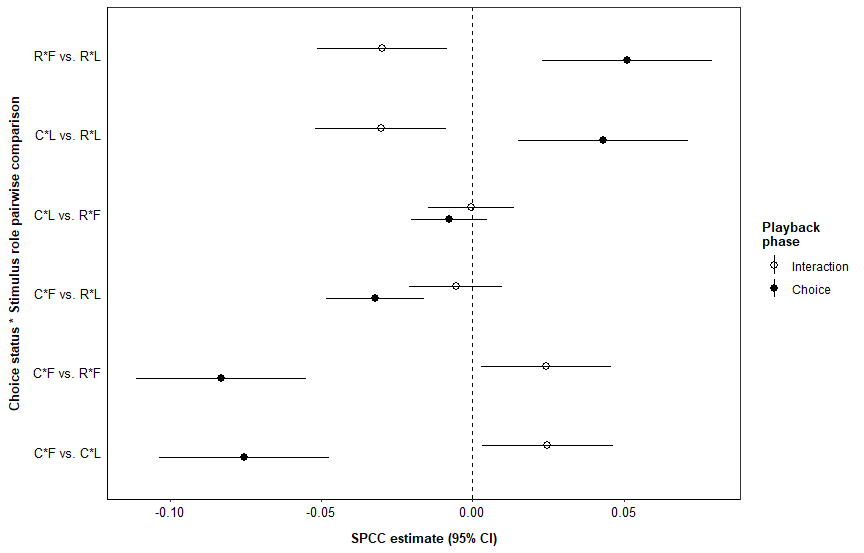

Supplement: S2 Fig — Model estimates and 95% confidence intervals of the LSMean difference in the spectrographic cross-correlation similarity between flock responses and stimulus calls in the interaction and choice phase of male-male trials. The figure shows each pairwise comparison of interactions between the choice status (C = chosen and R = not chosen) and role (L = leader and F = follower) of stimulus individuals. (TIF) [file pone.0252374.s010.tif]

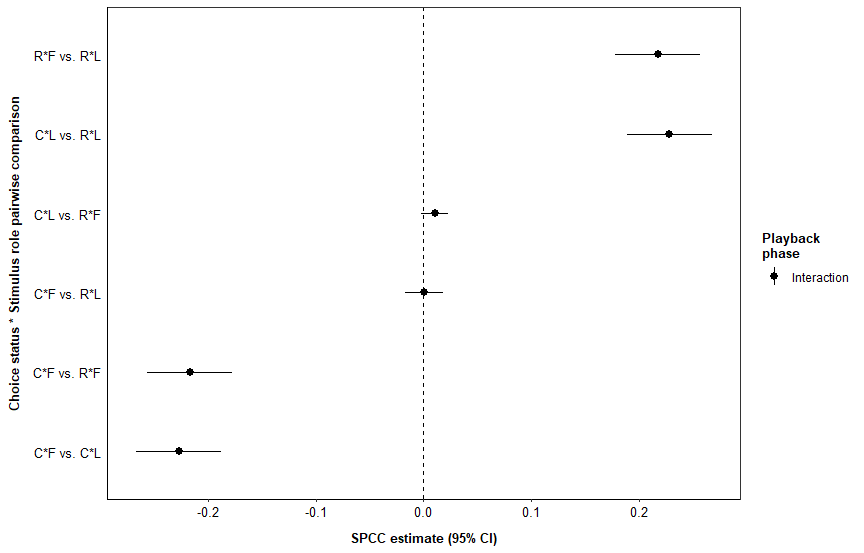

Supplement: S3 Fig — Model estimates and 95% confidence intervals of the LSMean difference in the spectrographic cross-correlation similarity between flock responses and stimulus calls in the interaction phase of male-female trials. The figure shows each pairwise comparison of interactions between the choice status (C = chosen and R = not chosen) and role (L = leader and F = follower) of stimulus individuals. (TIF) [file pone.0252374.s011.tif]

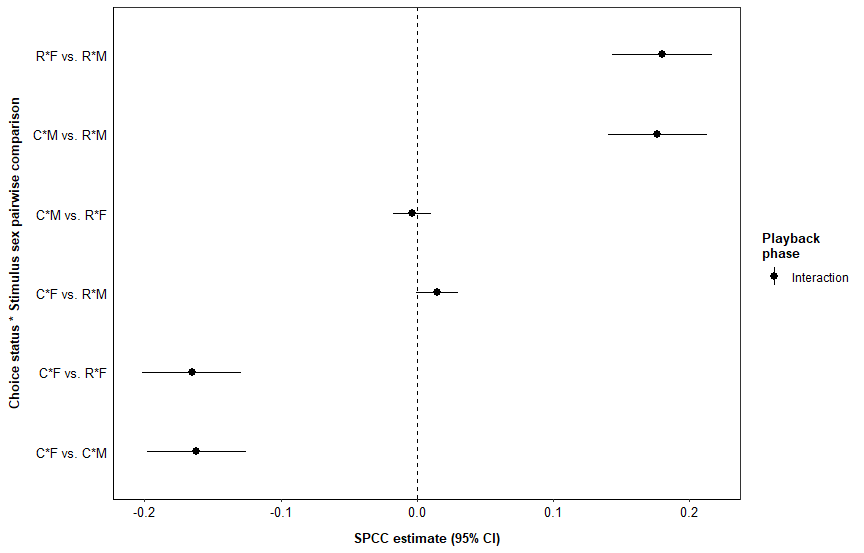

Supplement: S4 Fig — Model estimates and 95% confidence intervals of the LSMean difference in the spectrographic cross-correlation similarity between flock responses and stimulus calls in the interaction phase of male-female trials. The figure shows each pairwise comparison of interactions between the choice status (C = chosen and R = not chosen) and sex (M = male and F = female) of stimulus individuals. (TIF) [file pone.0252374.s012.tif]

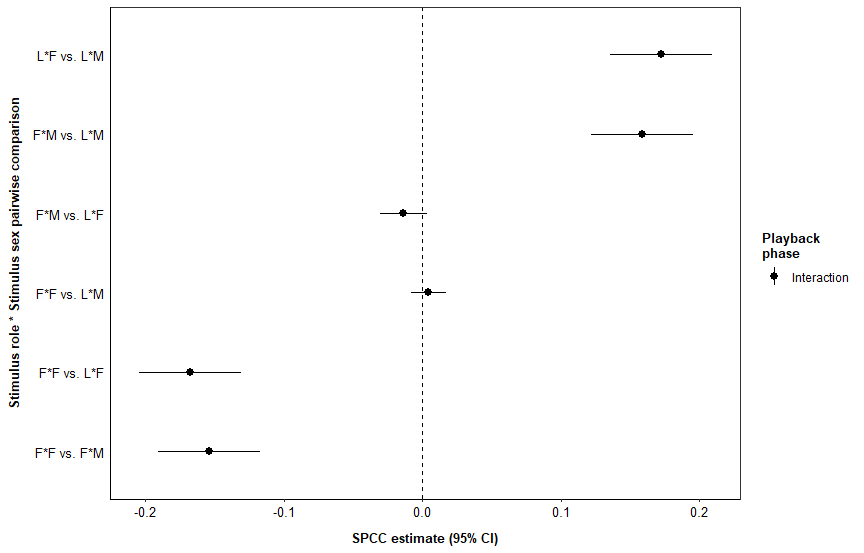

Supplement: S5 Fig — Model estimates and 95% confidence intervals of the LSMean difference in the spectrographic cross-correlation similarity between flock responses and stimulus calls in the interaction phase of male-female trials. The figure shows each pairwise comparison of interactions between the role (F = Follower and L = leader) and sex (M = male and F = female) of stimulus individuals. (TIF) [file pone.0252374.s013.tif]
