# Supplementary material for: Follow the leader? Orange-fronted conures eavesdrop on conspecific vocal performance and utilise it in social decisions
Source: PLoS One. 2021 Jun 9;16(6):e0252374. doi: 10.1371/journal.pone.0252374 (PMC8189466; doi:10.1371/journal.pone.0252374)
Supplement: S1 Model — The model for number of contact calls (response rate) that focal flocks emitted during (A) male-male and (B) male-female trials. Lower case letters show the fixed factors with second-order interactions shown as multiplications indicated with an asterisk. Capital letters correspond to any random factors added to the model. (DOCX) [file pone.0252374.s014.docx]

**A** Response rate = choice role + fusion type + flock size + LOCATION

**B** Response rate = choice role + choice sex + fusion type + flock size + choice role * choice sex + LOCATION
