# Supplementary material for: Follow the leader? Orange-fronted conures eavesdrop on conspecific vocal performance and utilise it in social decisions
Source: PLoS One. 2021 Jun 9;16(6):e0252374. doi: 10.1371/journal.pone.0252374 (PMC8189466; doi:10.1371/journal.pone.0252374)
Supplement: S2 Model — The model for spectrographic cross-correlation similarity between contact call responses from focal flocks and stimulus calls (response-playback similarity) in (A) male-male and (B) male-female trials. Lower case letters show the fixed factors with second-order interactions shown as multiplications indicated with an asterisk. Capital letters correspond to any random factors added to the model. (DOCX) [file pone.0252374.s015.docx]

**A** Response-playback similarity = choice status + stimulus role + fusion type + flock size + choice status * stimulus role

+ TRIAL NUMBER * RESPONSE ORDER

**B** Response-playback similarity = choice status + stimulus role + stimulus sex + fusion type + flock size + choice status * stimulus role + choice status * stimulus sex + stimulus role * stimulus sex + TRIAL NUMBER * RESPONSE ORDER
